# German Dentistry

**Published:** 1883-06

**Authors:** 


GERMAN DENTISTRY.

There are as yet, in the German Empire, 2,500 cities with a number of inhabitants varying between 5,000 and 50,000, without any dentist. This list includes only cities with 5,000 or more inhabitants, and does not notice those of smaller numbers. As an average, we find in Germany in cities above 50,000 one dentist to every 19,000 persons; in cities between 20,000 and 25,000, one dentist to every 20,000; in cities of 5,000 to 20,000 inhabitants dentists are found in the proportion of one to every 92,000 inhabitants, while in the smaller cities below 5,000, seven dentists are working hard to alleviate the pains due to teeth of six millions of people.—*Zahntechnische Reform.*

